# Using Virtual Reality to Provide Health Care Information to People With Intellectual Disabilities: Acceptability, Usability, and Potential Utility

**DOI:** 10.2196/jmir.1917

**Published:** 2011-11-14

**Authors:** Valerie Hall, Suzanne Conboy-Hill, Dave Taylor

**Affiliations:** ^1^Centre for Health ResearchUniversity of BrightonBrightonUnited Kingdom; ^2^Research and DevelopmentSussex Partnership NHS Foundation TrustHoveUnited Kingdom; ^3^Division of SurgeryImperial CollegeLondonUnited Kingdom

**Keywords:** Learning disabilities, intellectual disabilities, virtual reality, health information, participatory research, capacity to consent, presence

## Abstract

**Background:**

People with intellectual disabilities have poor access to health care, which may be further compromised by a lack of accessible health information. To be effective, health information must be easily understood and remembered. People with intellectual disabilities learn better from multimodal information sources, and virtual reality offers a 3-dimensional (3D) computer-generated environment that can be used for providing information and learning. To date, research into virtual reality experiences for people with intellectual disabilities has been limited to skill-based training and leisure opportunities within the young to mid age ranges.

**Objective:**

This study assessed the acceptability, usability, and potential utility of a virtual reality experience as a means of providing health care-related information to people with intellectual disabilities. We designed a prototype multimodal experience based on a hospital scenario and situated on an island in the Second Life 3D virtual world. We wanted to know how people of different ages and with varying levels of cognitive function would participate in the customized virtual environment, what they understood from being there, and what they remembered a week later.

**Methods:**

The study drew on qualitative data. We used a participatory research approach that involved working alongside people with intellectual disabilities and their supporters in a community setting. Cognitive function was assessed, using the Matrix Analogies Test and the British Picture Vocabulary Scale, to describe the sample. Participants, supported by facilitators, were video recorded accessing and engaging with the virtual environment. We assessed recall 1 week later, using a specialized interview technique. Data were downloaded into NVivo 8 and analyzed using the framework analysis technique.

**Results:**

Study participants were 20 people aged between 20 and 80 years with mild to severe intellectual disabilities. All participants were able to access the environment and voluntarily stayed there for between 23 and 57 minutes. With facilitator support, all participants moved the avatar themselves. Participants engaged with the scenario as if they were actually there, indicating cognitive presence. Some referred back to previous medical experiences, indicating the potential for experiential knowledge to become the foundation of new learning and retention of knowledge. When interviewed, all participants remembered some aspects of the environment.

**Conclusions:**

A sample of adults with intellectual disabilities of all ages, and with varying levels of cognitive function, accessed and enjoyed a virtual-world environment that drew on a health care-related scenario, and remembered aspects of it a week later. The small sample size limits generalizability of findings, but the potential shown for experiential learning to aid retention of knowledge on which consent is based appears promising. Successfully delivering health care-related information in a non-National Health Service setting indicates potential for delivery in institutional, community, or home settings, thereby widening access to the information.

## Introduction

People with intellectual disabilities have the poorest access to health care [1], which may be worsened by a lack of accessible health information. In the United Kingdom, the Mental Capacity Act [2] makes it a legal requirement for health professionals to ensure patients are given full information, to enable them to make their own decision about treatment. However, people with intellectual disabilities may have difficulties in taking in and retaining information in order to make that decision, and therefore they may not get the treatment they need, or they may get treatment they didn’t want. Even when they agree to treatment, if they do not fully understand what is going to happen to them, they may refuse to cooperate. This could be distressing for the person, his or her caregiver, and the health care staff, and may lead to a longer stay in hospital.

Normally, information leaflets and storybooks are used to provide health care information. However, a review of informed consent to health care interventions concludes that enhancing understanding may depend on the effort made to tailor the information to the abilities and needs of the individual with intellectual disabilities [3]. It is already known that learning in people with intellectual disabilities can be enhanced by using audio and video presentations [4,5]. Moreover, interactive multimedia technologies such as virtual reality provide opportunities for people to interact with virtual objects and events from everyday life, which can lead participants to feel that they are actually “there”—a subjective experience known as cognitive presence [6]. These techniques may help bridge the gap between information representation and experiential learning [7]. Virtual reality has been shown to support learning in people with intellectual disabilities in a variety of ways [8-11] and to provide a safe setting in which they can practice activities that might not be possible in the real world [12].

Gaming technology, which can enhance motivation, is being used increasingly to develop interventions that improve health knowledge and assist in health-related decision making for the general population [13]. Virtual reality gaming studies show that people with intellectual disabilities enjoy experiences that allow them to take control of their environment and succeed in activities that are usually inaccessible to them [14]. Use of Internet-based virtual reality environments, such as Second Life, is also increasing. These environments can be accessed from any location and offer unique and interactive ways to facilitate health care information, particularly when full advantage is taken of the experiential features [15].

Existing research conducted with people with intellectual disabilities, using virtual reality applications, mainly relates to skill-based training [9,10,16-18], rehabilitative skills [12,19], developing participation in exercise skills [20], or leisure activity [14], and is mostly undertaken with a younger group of people in institutional settings. Our study adds to existing knowledge because it reports on the acceptability, usability, and potential utility of virtual reality as a means of providing health care-related information to people with intellectual disabilities, and includes adult participants from the whole age range, including older people. Delivering health care-related information in a social setting indicates potential for its use in community or home settings, thereby widening peoples’ access to it. Importantly, we used a participatory research method, working alongside people with intellectual disabilities and their supporters to ensure their rights were recognized within the process and their experiences were properly represented [21].

## Methods

### Study Design

This exploratory study drew on qualitative data, to assess the acceptability, usability, and potential utility of a virtual reality experience to provide health care-related information to people with intellectual disabilities. We were interested in how people of different ages and with varying levels of cognitive function would participate in the customized virtual environment, what they understood from being there, and what they remembered a week later.

We wanted to make the research participatory by working alongside people with intellectual disabilities and their supporters. Therefore, we worked collaboratively with the Grace Eyre Foundation in Hove, East Sussex, UK, which is a registered charity, providing support and services in the community for people with intellectual disabilities. We also involved people with intellectual disabilities in the delivery of the study itself, taking care to remain mindful of the potential vulnerability of the participants, and the need to pay close attention to issues of recruitment and consent. The conduct of the research was overseen by a steering group, which included a person with intellectual disabilities and representatives from Grace Eyre. Not only did this provide a reference group within which to discuss and monitor ethical practice, but also members provided practical advice on the production of recruitment information.

The participants were video recorded while accessing and engaging with the virtual environment. Six volunteer psychology graduates, who had been given rudimentary instruction on the use of Second Life (http://secondlife.com/) and the navigational tools, acted as facilitators to the delivery of the virtual environment. They encouraged the participants to recognize landmarks, and to engage and experiment with various interactive elements contained within the environment.

We assessed each participant’s memory of the virtual environment exposure 1 week later using a modified cognitive interview technique. The cognitive interview is made up of two parts. In the first part, the participant is asked to recount as much as possible of the experience, without interruption. The interviewer then probes the information systematically, using contemporaneous notes. Nonleading questions target key items of information; other questions probe meaning. In the second part, participants are shown screenshots of the exposure and asked questions aimed at further prompting memory. This technique has been shown to increase the reporting of accurate recall from various population groups [22-24], particularly if, as in this study, staged events are used [25]. An experienced clinical psychologist conducted the modified cognitive interview, with a 1-week time lapse between exposure and interview to mirror usual clinical practice when assessing retention of information on which capacity to consent is assessed.

We chose to video record the participants accessing and engaging with the virtual environment, although we acknowledge the potential criticisms of observer bias in studies using observational data. However, the communication difficulties associated with our participant group, and their recognized tendency to agreement and compliance, precluded direct questioning through standard interview or questionnaire. Data triangulation, achieved through comparing the results of the observation analysis with cognitive interview data and a focus group validation of findings, served to support study credibility [26].

### Setting and Participants

The research was conducted at the Grace Eyre social center, which provided a well-resourced information technology suite. Undertaking the research at the center, rather than in a laboratory, allowed us to assess the potential for delivering the virtual environment in a real-life community setting. Moreover, it provided a sense of familiarity and security to the research participants. Participants were invited to bring their support workers, but only 7 chose to do so.

We recruited a convenience sample from people with intellectual disabilities who use the center. The sample comprised 20 people, 11 male and 9 female, between the ages of 20 and 80 years.

In order to describe the level of cognitive function in the sample, we used two minimally demanding screening tools: one for verbal material, and the other visual. The British Picture Vocabulary Scale (BPVS) assesses contextual receptive vocabulary and does not rely on speech or reading. The BPVS test is acceptable to adults with intellectual disabilities because there is minimal experience of failure. The Matrix Analogies Test (MAT) is a similar tool, requiring little language and no writing skills. Scoring for each is governed by clear, manualized criteria, and gives rise to raw, age-equivalent, and standard scores, from which an intelligence quotient can be estimated. From the analysis of the test results we determined that the sample consisted of people with low, medium, and high cognitive function within the intellectual disabilities range, with one exception indicated in the MAT score, a person with an autistic spectrum disorder (see Table 1). In addition, we observed other conditions such as Down syndrome.

Of the study participants, 16 had previously used a computer at Grace Eyre, but only 7 had medium and 1 had high levels of computer usage. Although the computers in the information technology suite were set up for use by people with intellectual disabilities, they had not previously been used to access Second Life. People with intellectual disabilities may have trouble operating multifunction control devices due to problems in remembering which device achieves which task, or they may experience fine-motor difficulties, which could leave them feeling frustrated and demotivated [27]. Using the equipment in the center, we undertook a small prestudy test to identify and rectify any preliminary problems with the navigational and interaction control devices used to access the virtual environment. No adjustments to the equipment were required.

**Table 1:** Levels of cognitive function within the sample of people with intellectual disabilities

BPVS^a^ age equivalent in years	Number of people in range (N = 20)	MAT^b^ standard score	Number of people in range (N = 20)
Low (0–4)	4	Low (≤49)	7
Medium (5–6)	14	Medium (50–75)	12
Highest (10–14)	2	Highest (76–100)	1

^a^ British Picture Vocabulary Scale.

^b^ Matrix Analogies Test.

#### Consent

We paid particular attention to obtaining consent in this potentially vulnerable population. People with intellectual disabilities helped us write the patient information sheet and consent forms (Multimedia Appendix 1, Multimedia Appendix 2). Posters were displayed in the Grace Eyre center with an invitation to contact a staff member for more information and support to decide whether to volunteer (Multimedia Appendix 3). Center staff sought initial consent from the participant but verbal consent was also sought immediately prior to each element of the study.

Approval to undertake the study was granted by the University of Brighton Research Ethics and Governance Committee. In addition, UK National Health Service Research Governance approval was granted.

### Virtual Environment Health Information Experience

**Figure 1:**
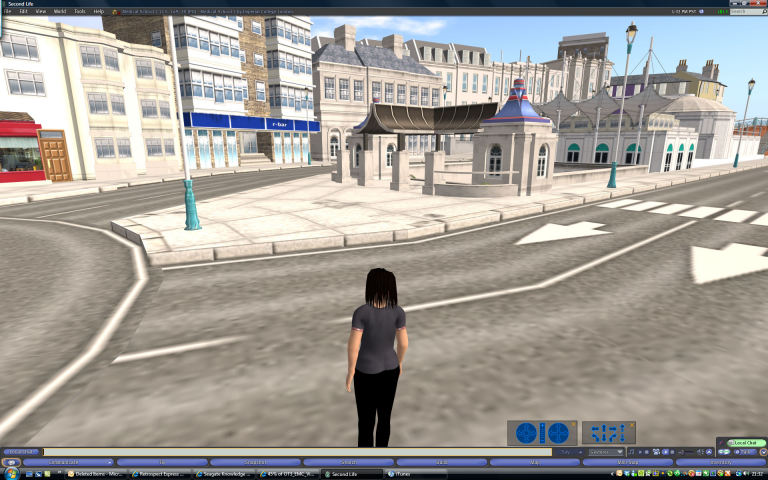
Screenshot of opening scene showing key landmarks and standard female avatar.

A virtual environment, representing a stylized hospital building and internal rooms, was designed by Imperial College London. The environment was hosted on a private Second Life server and was accessible to the study, but not to the public, on a desktop computer over the Internet. Access to the virtual environment was limited to authorized account holders. Authorized users could login and tour the environment while being represented as an animated avatar in male or female human form. Users were able to control their avatars’ movements using a computer keyboard’s arrow keys and a mouse. A realistic 3-dimensional rendering of the key landmarks around the hospital in Brighton was developed, and suitable streets that could be easily navigated were linked to these (see figure 1. A realistically animated ocean bounded the simulated environment, and distant views of cliffs and buildings were produced using large photographic images placed on the borders of the simulated space, rather like a film set. Two avatars were created with features that could be selected to provide a broad match with the participant, such as male or female, hair color, and ethnicity, plus a wheelchair if required.

**Figure 2:**
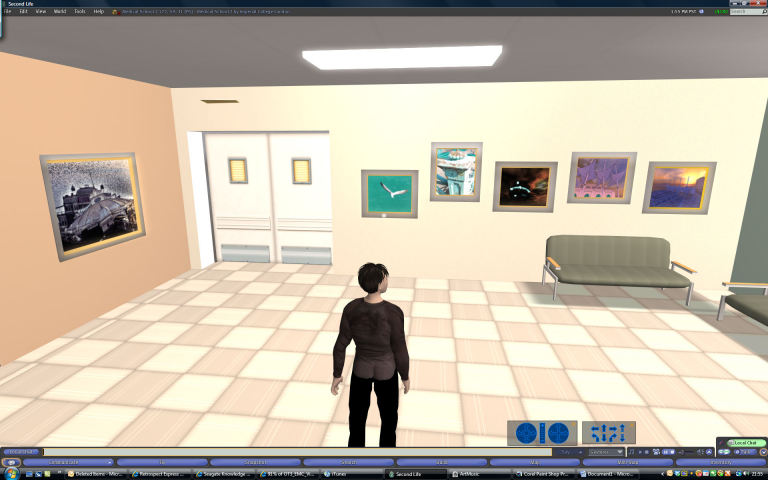
Screenshot of hospital waiting room.

Interiors of the hospital buildings were also replicated (see figure 2). The hospital scenario incorporated a programmed “nurse” robot, which was activated by participants taking a seat in a waiting room. The nurse communicated with the participants using preprogrammed text in a dialogue box and could be summoned by phone to a specific room in order to explain its purpose. In addition, the nurse offered a tour of the hospital, with the participants having the choice of being pushed on a hospital bed, using a wheelchair, or walking from room to room. The nurse robot was generated by a special version of the Second Life client software running on a virtual server in the Amazon “cloud” and controlled by a script that specified its responses and actions.

**Figure 3:**
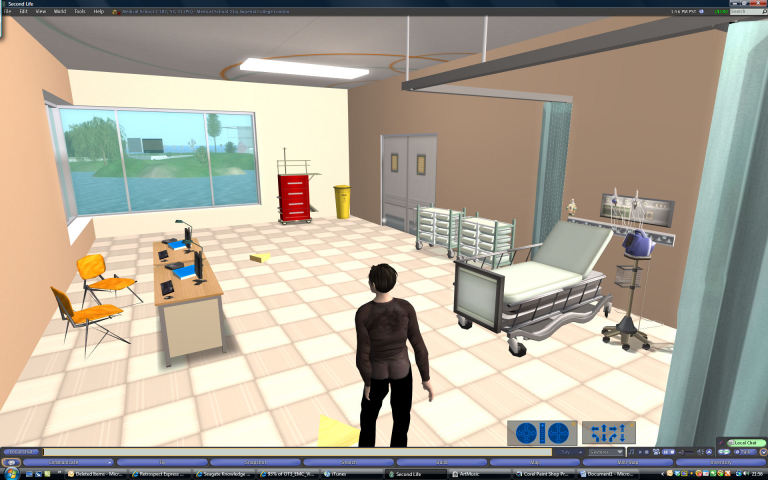
Screenshot of clinical examination room.

The hospital also included a clinical examination room, with a bed on which the participant was invited to lie, and could experience an interactive blood pressure machine and cuff (see figure 3, a preparation room, an operating room wherein the participant could lie on the operating table, and a recovery ward containing a static patient in a bed.

Other interactive hospital equipment was built into the rooms to provide a suitably realistic experience. All of these virtual-world objects were automated using programs written in the Second Life java-like scripting language (Linden Scripting Language). Multimedia Appendix 4 provides a video walk-through of the virtual environment.

### Data Capture, Production, and Analysis

The participants were video recorded while accessing and engaging with the virtual environment. A portable usability lab designed by the University of Brighton enabled us to capture audiovisual data in the community center. Two high-definition camcorders were strategically positioned: one to capture information about the physical use of computer equipment and navigational tools, and the other to capture the participants’ engagement with, and response to, the scenario on the computer screen. A video scaler and recorder captured concurrent images from the computer screen. Data from all three streams were merged using Apple iMovie 09 (Apple, Cupertino, CA, USA) video-editing software and downloaded into NVivo version 8 (QRS International, Doncaster, Australia), where an annotated account of key events in each exposure video was prepared.

Cognitive interviews were digitally recorded and transcribed verbatim. To determine the degree of accurate recall, a summary of each individual’s virtual environment experience was compared with the events they recalled in the cognitive interview. This was undertaken by the researcher who analyzed the video exposures, to reduce the risk of interviewer bias.

All data were entered into NVivo and analyzed the using the framework approach, which allows for both deductive and inductive analysis [28]. This approach involves a systematic process of sifting, charting, and sorting material according to key issues and themes, which is appropriately targeted toward providing “answers” in the form of greater illumination or understanding of the issues. As there were specific questions that we wanted to answer, the framework for analysis was formed from themes arising directly from these questions and from the literature (see Table 2).

Textual data were analyzed thematically, with key issues arising from the analysis being entered into a casebook matrix in order to compare results across different characteristics or within and across themes (Multimedia Appendix 5). For instance, from the cross-analysis we were able to report on whether the people who enjoyed the experience were more likely to have identified with the avatar.

**Table 2:** Major themes and issues underpinning the framework analysis

Major themes	Related issues
Temporality	Time taken to sit at computer, use keyboard, gain ability to move avatar, recognize scenario, identify with the avatar; overall time spent in Second Life and looking at the screen
Accessibility	Technical and physical barriers; chair and body position in relation to screen; moving and interacting in the virtual space; facilitators’ support of skills development
Context	Physical characteristics of user compared with avatar; recognition of scenario; facilitation style; role of support worker; culture of Grace Eyre; users’ previous experience of computers
Cognitive presence	Reaction to user interface, authentic scenario; identification with scenario; structure of scenario; visual cues; engagement with interactive activities; spontaneous recall of previous experience; relationship to avatar; emotional response to avatar actions; engagement with “nurse” robot; engagement with chat-based interaction; control; autonomy; enjoyment
Recall	Number of accurate statements, distortions, confabulations, and imported information

## Results

We set out to explore the acceptability, usability, and potential utility of virtual reality as a means of providing health care-related information to people with intellectual disabilities. All 20 participants completed the Second Life exposure and were interviewed, using the cognitive interview technique, within a 5-month period. The results from both these data sources are presented using the major theme headings outlined in Table 2.

### Temporality

All participants sat at the computer, began to engage with the virtual environment immediately, and maintained good concentration throughout, only disengaging for short tea or toilet breaks. Within 5 minutes of starting the virtual environment exposure, 18 people independently moved the avatar, with the other 2 taking up to 10 minutes to do so. A total of 17 participants recognized aspects of the Brighton scenario right away. All participants instantly noticed the avatar, and almost all of them thought that it was “pretending” to be them. They remained in the exposure voluntarily between 23 and 57 minutes, with the majority staying between 40 and 45 minutes.

### Accessibility

None of the participants had physical difficulty using the keyboard, although skill levels varied. Most of those who started with a low level of keyboard skills improved during the exposure. However, there were a small number whose skills remained underdeveloped throughout. With facilitator support, a lack of skills did not seem to inhibit either active engagement in the scenario or decisions as to where the avatar went and what it did.

The following extract from an annotated video record illustrates this observation:

[Participant] understands how to move the avatar but reluctant to do so on her own without support although later in this room her fingers hover over keys in readiness to press them before guided. Gets on bed with help. Stands on bed by mistake and finds this funny.

The facilitators acted mainly as guides but sometimes intervened when participants could have done things for themselves. The ability to move the avatar skillfully and autonomously meant that sometimes people concentrated more on movement than on the content of the virtual environment, as demonstrated in the following annotated video record:

Doesn’t know the purpose of the waiting room. Seeks help to move avatar onto seat. Not interested in the dialogue with the nurse or even acknowledging her presence other than to move the avatar to the next room.

### Context

Two avatars were created with features that could be chosen to provide a broad match with the participant, such as male or female, hair color, and ethnicity, plus a wheelchair if required. However, other than gender, and skin color to reflect ethnicity, none of the other features were used in the study.

As a result, only 4 participants looked similar to their avatar and therefore it is difficult to assess whether this affected identification with it.

Everyone recognized some aspects of the virtual environment, although not everyone identified the outside of the hospital or was aware of the purpose of all the rooms they visited. The operating room was the most likely to be recognized spontaneously during the virtual environment experience, followed by the waiting room. However, in the cognitive interview, participants remembered activities that occurred in the assessment room, such as the use of the blood pressure machine.

The facilitators were instructed to enable a nonthreatening experience using self-directed, informal, and playful strategies. A lighthearted approach is important because engaging emotionally with the virtual environment improves enjoyment, thereby aiding memory. However, observed styles ranged from enabling to directive across the participant group, and sometimes even within the individual’s virtual environment experience. An enabling style encouraged participants to go where they wanted and do what they wanted, but it did mean that the balance of the exposure was lost, and the person often did not spend time in every area. Additionally, it meant that they might not have had the opportunity to experience many of the activities. A more directive style often meant the participant spent time in all the areas, but it sometimes resulted in the facilitator taking over the controls or telling the participant where to visit, which lost some of the opportunities for playful engagement.

The ideal facilitation style appears to be one that enables access to all the opportunities available in the virtual environment but takes a negotiated approach to determining what the person will do in it, and how much help they need and want. However, it should also be gently directive in supporting the person to learn to move the avatar to the best of his or her ability, while spending sufficient time in each area. We term this assertive facilitation.

Support worker involvement was minimal, occasionally sharing jokes and enjoyment and offering encouragement. Only 1 participant needed continuous input from her support worker to help her stay focused.

Our participants were recruited from an organization that takes a positive stance toward its clients’ use of technology and provides a structured day that encourages focused activity. This positive environment and previous experience of using computers may have influenced the length of time participants voluntarily stayed at the keyboard and their willingness to engage in the experience.

### Cognitive Presence

Participants clearly knew that they were interacting with the virtual environment through a computer because they were using the keyboard and mouse to access it, but this did not detract from their engagement with the scenario. Several conditions promoted a sense of cognitive presence.

All participants identified with one or more of the virtual environment areas, with 17 recognizing Brighton sea front instantly. This led to a high degree of engagement, as the participants recalled previous experiences while in the virtual environment, expecting to see boats and to go swimming, and even expecting to be able to locate their own home. Most people were curious and explored the outdoor, as well as the indoor, environment. They tried to open doors in buildings to find out what was behind them; they rang doorbells and tried to sit on seats. Within the hospital component of the virtual environment they happily engaged in the programmed activities, such as having their blood pressure taken, or lying on the operating room table. One participant said “And I laid on a bed to see what it feels like when you do have an operation.”

More importantly, the experience also prompted them to recount prior experiences of hospital treatments, such as having blood or blood pressure taken, or x-rays, or more generally about being in hospital. When recognizing an x-ray machine, one participant said “I saw them before...when I had my hip done.”

These associations were so strong that some people recounted them in the interview a week later. The ability to extrapolate information from the virtual environment is important, as it indicates understanding of the scenario and provides a foundation for learning about it [29].

Full identification with the avatar did not seem to be important; only a few participants referred to the avatar as “I.” Others had a more superficial relationship with it, using it as a way of navigating through the scenario, with some expressing concern for what it was doing in potentially dangerous situations—for example, when crossing the road.

The technical performance of the nurse robot proved unpredictable during the virtual environment experience: some participants followed her and some verbalized recognizing her, but only one person wanted to talk to her. However, in the interview a week later, a large number of people mentioned a nurse and the patient in the bed.

Took him (the avatar)...um...yes...nurse going there—and I go—and (the nurse—dialogue spoken by the facilitator) asked me about it—about being in the hospital there.

There was some writing on the screen...um—I am better now and I will go home...yes—that is what the patient said.

This indicates the potential value of including a human-like “other” presence with a specific purpose appropriate to the scenario.

A total of 18 participants moved the avatar themselves or determined what it did. If the facilitator intervened too much, or control was taken away, some participants appeared to lose interest or confidence. In one example, a participant who chose to have his avatar pushed around the hospital on the bed epitomizes this loss of confidence. Prior to the bed tour the participant was attempting to move the avatar himself, but following the bed tour, he required continual prompting before he returned to his previous activity level. In another example, the facilitator takes over control of the keys, and the participant is observed losing concentration and starting to look round the information technology suite.

Maintaining control of the avatar allowed participants to satisfy their curiosity by going to look at things spontaneously. One participant asked “What’s that in there?” and, encouraged by the facilitator’s prompt “Let’s go and have a look—get a bit closer”, engaged in a dialogue about the use of the piece of hospital equipment.

The ability to move the avatar skillfully and autonomously was also linked with enjoyment. However, even those who had not improved their skills expressed enjoyment during the experience and also in the cognitive interview afterward, sometimes asking to repeat the virtual environment experience. For example, in response to the cognitive interview question “What else happened in the computer?” one participant with undeveloped skills responded “Dun a ’puter myself” and later in the interview the participant reiterated “Do it again,” and yet later “Do it again—what date?”

Even when skills were underdeveloped, participants still wanted to navigate the avatar themselves and expressed enjoyment at the result, prompting exclamations such as:

Because you can look up the hospital.You can press what you can do on those little—thing—pointer things. On the computer. You can. Yes I enjoyed looking on the computer.

Judged through observations of their facial expressions and body language during the exposure, 17 participants demonstrated enjoyment—for instance, smiling, leaning forward into the screen, laughing and pointing to elements on screen, and commenting and making jokes. Although we cannot say whether the other 3 participants enjoyed the experience because their expressions remained neutral throughout, they did not show any signs of physical or emotional agitation such as rocking, stereotypy, or distractibility. In fact, all 3 stayed in the virtual environment voluntarily for over 30 minutes.

From the above it can be seen that enjoyment was linked to the recognition of the scenario, the sense of achievement in moving or directing the avatar, or engagement in the activities, which sometimes stimulated wonder and amusement. When asked to explain, in the cognitive interview, why she had said the experience was quite good, one participant said:

Well, going into hospital and look around. They give you confidence and then you won’t be frightened when you do go in.

### Recall

Participants were interviewed 1 week after the virtual environment experience. The cognitive interview is made up of two parts. In the first part, the participant was asked to recount as much as possible of the experience, without interruption. Nonleading questions targeted key items of information and asked—for instance, “You said you stood in the operating theatre, tell me what you saw in the operating theatre.” Other questions probed meaning, as in “You said there was an operating table; tell me what that’s for, what happens there.” In the second part of the interview, participants were shown screenshots of the exposure and were asked questions aimed at further prompting memory (for examples of screenshots see Figure 1, Figure 2, and Figure 3).

All participants reported some accurate memories, but the amount varied. Both parts of the interview elicited some made-up (confabulatory) information, or information that was added from their own experiences. This was significantly higher when memory was prompted by screenshots, with some participants “remembering” information about parts of the scenario that they had not visited. Although the inclusion of confabulated information is not unusual in the nonintellectual disabilities population, the increased reporting in the screenshot section indicates that the interview procedure needs to be revised. However, although our primary aim was to test for accuracy, it is important to bear in mind that the confabulated information was often based on the participant’s own personal experiences, indicating that they had an understanding of what was in the virtual environment and demonstrating potential for building on this knowledge.

### Validation of the Findings

At the end of the study, 2 months after the cognitive interview, 8 participants volunteered to take part in a 35-minute structured focus group to validate the results. The clinical psychologist and another member of the research team with experience of working with marginalized groups led the participants through the findings. To enhance the credibility of this process, we invited one of the steering group members, a person with intellectual disabilities, to observe the focus group.

The participants confirmed that the results matched their perceptions of the virtual environment experience. They all offered unprompted comments about various aspects of the exposure, and many were keen to be involved in further stages of the virtual environment development, with some suggesting other things that could be added to the scenario. At the end of the focus group, our steering group member spontaneously commented that he was impressed that the participants were still “buzzing” about the experience 2 months later.

## Discussion

The study found that adults with intellectual disabilities of all ages, and with various levels of cognitive function, could access and enjoy a virtual environment that drew on a health care-related scenario. They engaged with the scenario as if they were actually there, which encouraged them to talk about previous experiences. This indicates a potential for experiential knowledge to become the foundation of new learning about a health care-related scenario. All participants were able to remember some aspects of the virtual environment when interviewed a week later.

We were surprised by the degree of concentration and engagement shown by the participants. One of the influencing factors might have been the high degree of scenario recognition, through which the participants appeared to exhibit the subjective sensation of feeling and behaving as if they were actually there [30]. This engagement with the scenario is akin to the concept of cognitive presence, the predeterminants of which have been attributed variously to facilitation by technological equipment [7,31], or its psychological [32] or multidimensional nature [6]. Heeter’s [6] 3-dimensional model, consisting of personal presence (the sense of being there), social presence (reaction to other beings in the virtual environment), and environmental presence (the extent to which the environment appears to know that the person is there), best reflects the way we designed the environment. Our observations of the participants’ responses to the environment, in terms of their relationship to the nurse and patient, plus the interactive activities, support the relevance of the components of Heeter’s model. However, our participants were also aware of the need to use navigational tools to access the experience, but this did not seem to undermine their sense of being there, contrary to other authors’ suggestions that the sense of presence is greater when the mediation process remains unnoticed by the users [33]. Heeter [34] argues that other factors affect presence in nonmediated situations, and further consideration of these might help conceptualization of mediated presence. Thus, our findings support Thornson and colleague’s [32] arguments for presence as a phenomenon occurring in the mind, rather than in the specific technology. Human factors may also determine a person’s tendency to experience the cognitive state of presence [35]. Although the factors, described by Thornson et al [32] as empathy, spatial orientation, cognitive involvement (both passive and active), ability to construct mental models, and introversion, were identified using questionnaires with college graduates, they demonstrate some resonance with the observations made of the participants in this study. However achieved, the subjective experience of being there prompted participants in this study to remember previous experiences of medical treatments and visits to a hospital, which has the potential to provide the foundation for new learning, to open up an opportunity for clinical dialogue in order to elicit additional clinical information, and to assess psychosocial concerns [36].

These findings have implications for the mode of information delivery required to enhance the assessment of capacity to consent in people with intellectual disabilities and other marginalized groups. Del Carmen and Joffe [37] indicate five elements necessary for valid informed consent: voluntarism, capacity, disclosure, understanding, and decision. Assessment of capacity depends on the quality of the information provided, and this study has examined the feasibility of using a virtual environment as a way of *disclosing* information to people with intellectual disabilities in a way that enables them to *understand* the information and its relevance to their own situation. It is clear that the people in this study could access the virtual environment, engage with it for long enough to understand what it represented, and remember information about it a week later, mirroring the time lapse between giving information and interviewing to assess capacity that occurs in actual practice.

Much of the research regarding consent in vulnerable populations relates to ability to recall information [38,39] or to make decisions [40]; however, there are also issues of ongoing consent, which have yet to be addressed [41]. Using a virtual environment to provide information to enable valid consent means it could be accessed and used freely, not only as a way of providing information on which the individual is assessed to have capacity to consent, but also, after initial consent, to ensure ongoing consent. Similarly, the opportunity to practice being a patient before coming into hospital may provide an increased sense of control over health care experiences [15].

In this study, psychology graduates facilitated access to the health care information and, although they had limited expertise in working with people with intellectual disabilities and no previous knowledge of Second Life, they needed little training to help participants access and navigate in Second Life. While we have commented on differing facilitation styles and speculated on how they might have influenced the participants’ experience, this is largely because the virtual environment prototype was exploratory, related to a nonspecific health information event, and included greater opportunities for divergence from the health information purpose. A virtual environment designed to deliver health care information on a specific treatment would be more tightly structured, and therefore the balance between enabling and directive facilitation would change, depending on the purpose of its use and the role of the person providing the facilitation. This study indicates that the virtual environment could be delivered not only in health institutions and community environments, but also by caregivers and support workers as well as health care professionals. For instance, a physician could use the virtual environment initially to explain a potential treatment scenario, and this may require more directive facilitation, whereas a nurse or support worker may use the virtual environment to help understanding and reinforce the information. Additionally they might use it to help the person rehearse what might happen in a treatment situation, using a less directive approach, enabling the person to talk about previous experiences and possible concerns [42]. However, further work is required to ascertain the degree of skill required to avoid overcontrol and disengagement, or low control and inadequate exposure and therefore to maximize engagement. Our proposed next phase of the research intends to address this.

### Limitations

Our participants were recruited from an organization that takes a positive stance toward its clients’ use of technology and provides a structured day that encourages focused activity. Our small sample was recruited exclusively from this proactive organization, which precludes us from generalizing the findings to a wider population of people with intellectual disabilities. Moreover, we recognize that the scenario developed for this study was specifically tailored to our participants, which may have had a positive impact on the findings. While it may not be possible to customize future health care information experiences quite so specifically, new and emerging technological techniques and platforms, such as consumer-oriented 3-dimensional modeling, augmented reality, and high-fidelity 3-dimensional scanning, could enable some degree of “recognition.”

We argued earlier that potential criticisms of interpreter bias regarding observation data have been offset by triangulating these data against the verbal responses made in the cognitive interview and by validating the findings in the focus group. However, we acknowledge that there may still have been some subjective bias, particularly when commenting on visual or behavioral expressions of enjoyment. In future work we would consider developing a specialized interview technique for gaining verbal and nonverbal accounts of participants’ views.

### Conclusion

Our study clearly demonstrates the potential for using virtual reality to provide health care-related information to people with intellectual disabilities. People with intellectual disabilities engaged with a health-related virtual environment experience as if they were actually there, which prompted them to talk about previous health care experiences. It also provided an opportunity for them to practice being patients, potentially providing more information about themselves and their worries, which could lead to an increase in confidence in treatment situations. Although we have not yet tested the effectiveness of the virtual environment model against existing 2-dimensional health care information delivery methods, the potential for experiential learning indicated in our study appears promising. Furthermore, successfully delivering health care-related information in social settings indicates potential for use in a variety of settings. Moreover, the study indicates the potential for several health-related applications such as use by physicians to explain treatments, or by nurses and support workers to help understanding and enable the person to rehearse what might happen in a treatment situation. Importantly, the opportunity to revisit the information-giving scenario offered by virtual environments may provide a way of addressing issues of ongoing consent.

Our study is the first step on a path to providing effective health information to people with intellectual disabilities, and we have learned a great deal by taking it. Our next step is to further develop the prototype with help from volunteers from our participant group. We will then test it out in a larger and more diverse population of people with intellectual disabilities and in a range of settings, drawing on the lessons learned in this exploratory study.

## Multimedia Appendix 1

Patient information sheet.

## Multimedia Appendix 2

Consent form.

## Multimedia Appendix 3

Recruitment poster.

## Multimedia Appendix 4

Video walk-through of the virtual environment.

## Multimedia Appendix 5

NVivo 8 casebook analysis matrix showing attributes.

## Abbreviations

BPVS: British Picture Vocabulary Scale
MAT: Matrix Analogies Test

## References

[1] (2007). Mencap.

[2] UK HM Government (2007). UK National Archives.

[3] Goldsmith L, Skirton H, Webb C (2008). Informed consent to healthcare interventions in people with learning disabilities--an integrative review. J Adv Nurs.

[4] Dunn A, Kroese BS, Thomas G, Mcgarry A, Drew P (2006). “Are you allowed to say that?” Using video materials to provide accessible information about psychology services. Br J Learn Disabil.

[5] Broughton S (2002). A review of the literature: interventions to maximize capacity to consent and reduce anxiety of women with learning disabilities preparing for a cervical smear test. Health Serv Manage Res.

[6] Heeter C (1992). Being there: the subjective experience of presence. Presence (Camb).

[7] Winn W (1993). Human Interface Technology Laboratory, Washington Technology Center, University of Washington.

[8] Parsons S, Mitchell P (2002). The potential of virtual reality in social skills training for people with autistic spectrum disorders. J Intellect Disabil Res.

[9] Cromby JJ, Standen PJ, Newman J, Tasker H (1996). Successful transfer to the real world of skills practised in a virtual environment by students with severe learning difficulties. Proceedings.

[10] Cromby JJ, Standen PJ, Brown DJ (1996). The potentials of virtual environments in the education and training of people with learning disabilities. J Intellect Disabil Res.

[11] Alm N, Arnott JL, Murray IR, Buchanan I (1998). Virtual reality for putting people with disabilities in control.

[12] Schultheis MT, Rizzo AA (2001). The application of virtual reality technology in rehabilitation. Rehabil Psychol.

[13] Reichlin L, Mani N, McArthur K, Harris AM, Rajan N, Dacso CC (2011). Assessing the acceptability and usability of an interactive serious game in aiding treatment decisions for patients with localized prostate cancer. J Med Internet Res.

[14] Yalon-Chamovitz S, Weiss PL (2008). Virtual reality as a leisure activity for young adults with physical and intellectual disabilities. Res Dev Disabil.

[15] Beard L, Wilson K, Morra D, Keelan J (2009). A survey of health-related activities on second life. J Med Internet Res.

[16] Rose FD, Brooks BM, Attree EA (2002). An exploratory investigation into the usability and usefulness of training people with learning disabilities in a virtual environment. Disabil Rehabil.

[17] Brooks BM, Rose FD, Attree EA, Elliot-Square A (2002). An evaluation of the efficacy of training people with learning disabilities in a virtual environment. Disabil Rehabil.

[18] Groenewegen S, Heinz S, Frohlich B, Huckauf A (2008). Virtual world interfaces for special needs education based on props on a board. Comput Graph.

[19] Standen PJ, Brown DJ (2005). Virtual reality in the rehabilitation of people with intellectual disabilities: review. Cyberpsychol Behav.

[20] Lotan M, Yalon-Chamovitz S, Weiss PL (2009). Lessons learned towards a best practices model of virtual reality intervention for individuals with intellectual and developmental disability.

[21] Walmsley J (2004). Involving users with learning difficulties in health improvement: lessons from inclusive learning disability research. Nurs Inq.

[22] Memon A, Bull R (1991). The cognitive interview: its origins, empirical support, evaluation and practical implications. J Commun Appl Soc Psychol.

[23] Milne R, Bull R (2002). Back to basics: a componential analysis of the original cognitive interview mnemonics with three age groups. Appl Cogn Psychol.

[24] Conboy-Hill S (2006). Vulnerable adults: assessing capacity to consent. Clin Psych Forum.

[25] Köhnken G, Milne R, Memon A, Bull R (1999). The cognitive interview: a meta-analysis. Psychol Crime Law.

[26] Guba E, Lincoln Y (1989). Fourth Generation Evaluation.

[27] Standen PJ, Brown DJ, Anderton N, Battersby S (2006). Systematic evaluation of current control devices used by people with intellectual disabilities in non-immersive virtual environments. Cyberpsychol Behav.

[28] Richie J, Spencer L (2002). Qualitative data analysis for applied policy research. Huberman AM, Miles M, editors. The Qualitative Researcher’s Companion.

[29] Knowles M (1980). The Modern Practice of Adult Education: From Pedagogy to Andragogy.

[30] Barfield W, Zeltzer D, Sheridan T, Slater M (1995). Presence and performance within virtual environments. Barfield W, Furness III T, editors. Virtual Environments and Advanced Interface Design.

[31] Biocca F, Levy MR (1995). Communication in the Age of Virtual Reality.

[32] Thornson CA, Goldiez BF, Le H (2009). Predicting presence: constructing the tendency toward presence Inventory. Int J Hum Comput Stud.

[33] Lombard M, Ditton T (2006). At the heart of it all: the concept of presence. J Comput Mediat Commun.

[34] Heeter C (2003). Reflections on real presence by a virtual person. Presence (Camb).

[35] Slater M, Guger C, Edlinger G, Leeb R, Pfurtscheller G, Antley A, Garau M, Brogni A, Friedman D (2006). Analysis of physiological responses to a social situation in an immersive virtual environment. Presence (Camb).

[36] Schachter M, Fins JJ (2008). Informed consent revisited: a doctrine in the service of cancer care. Oncologist.

[37] del Carmen MG, Joffe S (2005). Informed consent for medical treatment and research: a review. Oncologist.

[38] Festinger DS, Ratanadilok K, Marlowe DB, Dugosh KL, Patapis NS, Dematteo DS (2007). Neuropsychological functioning and recall of research consent information among drug court clients. Ethics Behav.

[39] Festinger DS, Dugosh KL, Croft JR, Arabia PL, Marlowe DB (2010). Corrected feedback: a procedure to enhance recall of informed consent to research among substance abusing offenders. Ethics Behav.

[40] Palmer BW, Savla GN (2007). The association of specific neuropsychological deficits with capacity to consent to research or treatment. J Int Neuropsychol Soc.

[41] Johnson-Greene D (2008). Evolving standards for informed consent: is it time for an individualized and flexible approach?. Bersoff DN, editor. Ethical Conflicts in Psychology, 4th edition.

[42] Gorini A, Gaggioli A, Vigna C, Riva G (2008). A second life for eHealth: prospects for the use of 3-D virtual worlds in clinical psychology. J Med Internet Res.

