# Supplementary material for: Using Virtual Reality to Provide Health Care Information to People With Intellectual Disabilities: Acceptability, Usability, and Potential Utility
Source: J Med Internet Res. 2011 Nov 14;13(4):e91. doi: 10.2196/jmir.1917 (PMC3222201; doi:10.2196/jmir.1917)
Supplement: Supplementary file 1 [file jmir_v13i4e91_app1.pdf]

Imperial College London

University of Brighton

Sussex Partnership NHS Foundation Trust

grace eyre

Study about consent and people with learning disabilities using a 'computer hospital'*

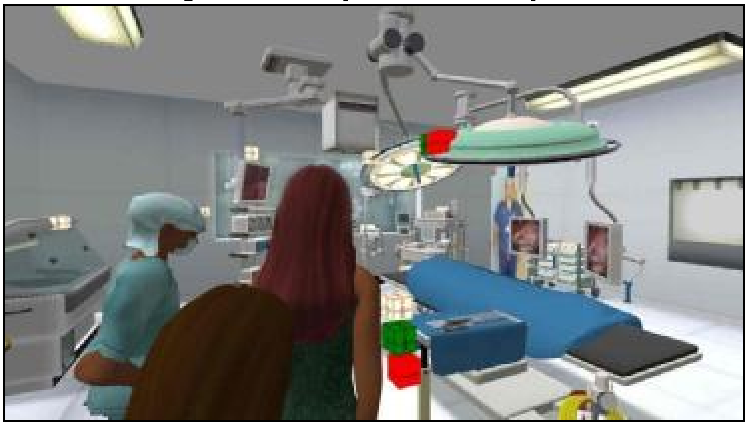

What is the study for? To find out if using a hospital on a computer helps people with learning disabilities to understand what might happen if they go into hospital.

What will we be trying to find out? How people with learning disabilities use a hospital on a computer to find out about going into hospital.

Why have I got this information sheet? You looked at our poster. Our poster asked if you'd like to be in our study and you said yes.

What will I have to do to be in the study? You'll have to do **3** things:

1. You'll come to the Grace Eyre centre and use the computer hospital. Someone from the study or your carer will help you use the computer.

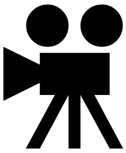

We will film you using the computer. It will take about an hour.

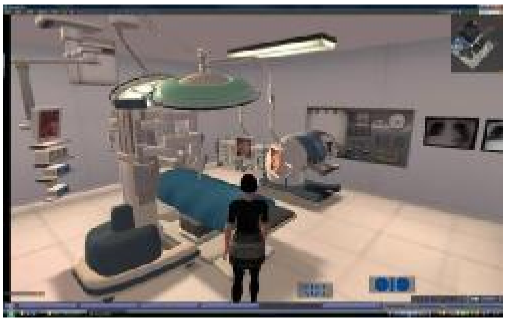

* Full title: 'Towards an NHS toolkit for obtaining consent for hospital admission from adults with learning disability incorporating a novel virtual world experience and a specialist interview technique.'

2. You'll come back to the Grace Eyre centre a bit later and meet Suzanne. She will ask you to tell her all about the computer hospital. It's not a test, it doesn't matter if you can't remember everything. We will record your voice while you are talking to Suzanne. It will take about an hour.

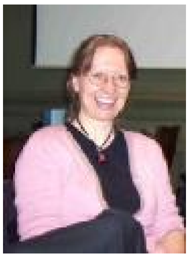

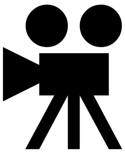

3. Someone else from the study will do some puzzles with you. You'll look at pictures and words and say what they mean or make patterns with them. Doing the puzzles will help us know if you prefer looking or talking.
It will take about an hour.

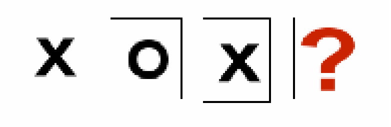

Do I have to take part? No, you can say no right now. If you say yes but change your mind, you can stop any time. You don't have to say why.

Who will know what I have said if I take part? Only the people running the study will know what you said. We need to tell other people about what we found out so we may show bits of the film or use some of the things you said. We will not give anybody's names when we do that.

We will keep all the films and tape recordings in a locked cupboard so they are private.

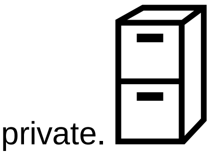

They will be destroyed, cut up, after ten years.

If I take part, will it hurt me or frighten me? Nothing in the study is planned to scare or hurt you.

If you do get upset at anytime, we will help and you can stop. We can also help you talk to someone outside the study if you want to do that.

What will I get for taking part? To say thank you for helping us we will give you a CD or a DVD that you choose. We will pay for you and your carer to travel to the Grace Eyre centre.

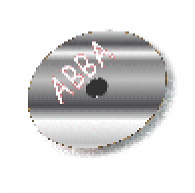

Will you tell me about what the study finds out? Yes. At the end of the study we will come back and tell everyone who helped us with the study what we found out. No one will know that you took part in the study or what you said unless you tell them yourself.

Who is doing this study?

It's being done by

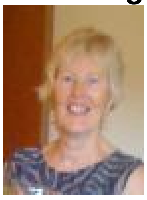

Professor Valerie Hall (University of Brighton),

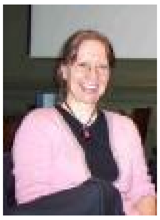

Dr Suzanne Conboy-Hill (Consultant Psychologist, Sussex Partnership NHS Foundation Trust),

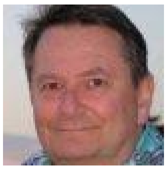

Dave Taylor (Imperial College London)

and Eva Jarvis (Grace Eyre Foundation).

The study has been looked at by the University of Brighton Research Ethics Committee to make sure that we are doing things properly.

If you want to know more about the study please let Professor Valerie Hall know. Her telephone number at the University of Brighton is (01273) 644015.

If you are unhappy about anything in the study you can talk to Angie Hart on 01273 644051, she works at the University but she is separate from the research.

If you would rather talk to someone who is not at the University you can ring: Dave Matthews at Grace Eyre Tel. 01273-201908

Or contact the Advocacy Organisations:

Interact	*Graham Lee*	*Tel: 01273-422971*
Speak Out	*Liz, Sarah, Ingrid*	*Tel: 01273-421921*
