# Supplementary material for: Using Virtual Reality to Provide Health Care Information to People With Intellectual Disabilities: Acceptability, Usability, and Potential Utility
Source: J Med Internet Res. 2011 Nov 14;13(4):e91. doi: 10.2196/jmir.1917 (PMC3222201; doi:10.2196/jmir.1917)
Supplement: Supplementary file 2 [file jmir_v13i4e91_app2.pdf]

Participant Consent Form
Study about consent and people with learning disabilities using a ‘computer hospital’

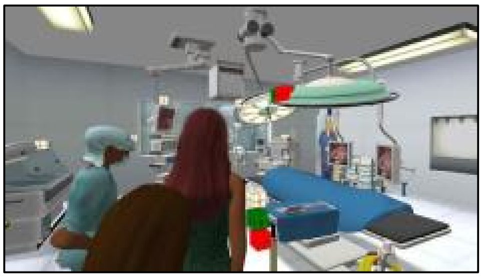

- I AGREE to looking at the computer hospital, answering the questions about it, and doing the puzzles.
- I AGREE to having a film made while I look at the computer hospital.
- I AGREE to having my voice recorded when I answer questions about it.
- I AGREE that the people running the study can look at the film and hear my answers to the questions and puzzles.
- I know that I can say I want to stop at any time and I don’t have to say why.
- I know that what I say will be used in the study but my name won’t be printed or mentioned.
- I know that the study will keep my answers and my films private 🗄 so no-one else can see them. They will be destroyed, cut up, " after the study is finished[i].
- I know that the study will be written up for lots of people to read 🕮 and to talk about at conferences. People I don’t know will hear about my answers and see bits of films but they won’t know who I am.
- I AGREE and SAY YES, **R** to all these things.

Participant name and signature and date where possible and meaningful

____

Witness name and signature confirmation and date where oral consent only is given

____

Researcher name and signature and date

____

[i] This means ten years after the study
