# Supplementary material for: Using Virtual Reality to Provide Health Care Information to People With Intellectual Disabilities: Acceptability, Usability, and Potential Utility
Source: J Med Internet Res. 2011 Nov 14;13(4):e91. doi: 10.2196/jmir.1917 (PMC3222201; doi:10.2196/jmir.1917)
Supplement: Supplementary file 3 [file jmir_v13i4e91_app3.pdf]

We need your help with a study!

What's it about?

- **We've built a hospital in a computer**

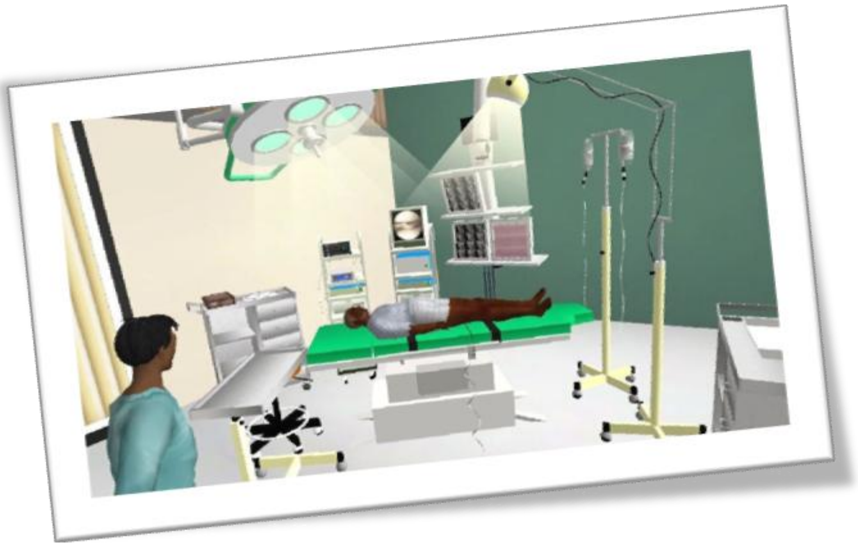

....

Yes really!

- We'd like you to look at it for us and tell us what you think.

Where do I have to go?

- We're doing the study in the computer room here

Will it cost me anything?

- No, not at all! In fact we'll give you something for helping us. A CD or DVD that you can choose.

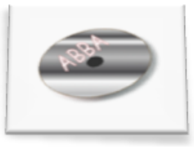

Who do I ask about doing the study?

- Eva Jarvis, Chris Bland, or Dave Matthews
